# Affective peculiarities in patients with drug-resistant epilepsy

**DOI:** 10.1192/j.eurpsy.2025.1816

**Published:** 2025-08-26

**Authors:** G. Carausu, N. Doten

**Affiliations:** 1 Department of Mental Health, Medical Psychology and Psychotherapy, State University of Medicine and Pharmacy, Chisinau; 2 National Centre of Epileptology, Institute of Emergency Medicine, Chișinău; 3 State University of Moldova; 4 Laboratory of Neurobiology and Medical Genetics, USMF Nicolae Testemitanu, Chisinau, Moldova, Republic of

## Abstract

**Introduction:**

According to WHO data, currently more than 50 million people suffer from epilepsy, about 30% of these people are diagnosed with drug-resistant epilepsy. Drug-resistant epilepsy involves multiple psychological problems such as cognitive impairment, emotional and behavioural disorders, which contribute to a reduced quality of life.

**Objectives:**

The aim of our study was a comparative analysis of the affective peculiarities of patients with drug-resistant epilepsy and patients with well-controlled epilepsy.

**Methods:**

The research took place during 2020-2023 within the National Centre of Epileptology, Chisinau, Republic of Moldova. The study was conducted on a group of 102 subjects with epilepsy (aged 18-62 years), including 62 subjects with drug-resistant epilepsy and 40 subjects with well-controlled epilepsy. We applied the Beck Depression Inventory (BDI) and Hamilton Anxiety Rating Scale (HAM-A). Statistical methods used are analysis of frequencies and percentage values, comparison of means by descriptive statistics, determination of Pearson correlation coefficients, difference of means by independent T-test.

**Results:**

According to the research results, depression is present in 43% of subjects, of which 23% in subjects with well-controlled epilepsy and 56% in those with drug-resistant epilepsy. Anxiety is present in 44 % subjects with epilepsy, of which 53% are subjects with drug-resistant epilepsy and only 33% with drug-controlled epilepsy. Statistical results reveal significant differences in the manifestation of depression (t=3.50 and p=0.001) and anxiety (t=3.09 and p=0.003) in subjects with drug-resistant epilepsy compared to those with well-controlled epilepsy. At the same time, no significant gender differences were found, but we find that women with drug-resistant epilepsy are 3.3 times more depressed and 2.6 times more anxious compared to women with well-controlled epilepsy. Divorced, non-employed, secondary technical education subjects with epilepsy of unknown aetiology are most affected by depression and anxiety, while subjects with higher education and those employed are more protected from suffering from anxiety and depression. As the disease progresses over time and the number of epileptic seizures increases, so does the level of depression and anxiety.

**Conclusions:**

Patients with drug-resistant epilepsy have significantly higher values for the depression and anxiety, compared to subjects with well-controlled epilepsy. Depression and anxiety were found in more than half of the drug-resistant subjects and twice less in subjects with well-controlled epilepsy.

**Disclosure of Interest:**

None Declared

